# Which people are most affected by changes to data linkage methodology? An exploration of patient, organisational and spatiotemporal characteristics in administrative hospital data in England

**DOI:** 10.23889/ijpds.v8i2.2235

**Published:** 2023-09-14

**Authors:** Tony Stone, Katie Harron

**Affiliations:** 1 Great Ormond Street Institute of Child Health, University College London, London, United Kingdom; 2 School of Health and Related Research, University of Sheffield, Sheffield, United Kingdom

## Objectives

In 2021, NHS Digital changed the process used to link records belonging to the same person across and within data collections. Our objectives were to identify patient, organisational and spatiotemporal characteristics associated with records impacted by this change and the implications for researchers using this data.

## Methods

We used an observational cohort study of patients, aged 55 or less, with a secondary care contact recorded in any of the NHS Digital (now part of NHS England) curated Hospital Episode Statistics (HES) datasets between April 1997 and March 2021. We compared clusters of records assigned to each patient using the HES ID (old methodology using a three-step deterministic algorithm) and the Person ID (new methodology using a master patient spine). We used multivariable logistic regression to identify patient, organisational and spatiotemporal (such as area-level deprivation and year of first contact) characteristics associated with patients whose cluster had changed.

## Results

Of 88 million hospital records in 2019, there were 18,968,711 distinct HES IDs and 18,717,142 distinct TPIs. Of the 12,701,169 HES IDs with more than one record, 145,948 (1.1%) were split into multiple Person IDs. Of the 12,999,671 Person IDs with more than one record, 483,091 (3.7%) were associated with two or more merged HES IDs. We will present an analysis using data covering the period April 1997 to March 2021 - 1.25 billion records - and present the characteristics associated with changes between linkage methods.

## Conclusion

Our findings indicate that this change consolidated clusters, resulting in fewer distinct individuals in the data. Our findings will inform researchers about which groups of individuals are most likely to be affected by changes to linkage methodology. This is vital for understanding potential sources of bias due to linkage error.

